# Low levels of adropin are associated with acute kidney injury after decongestion in patients with acutely decompensated heart failure

**DOI:** 10.1016/j.jmccpl.2025.100302

**Published:** 2025-05-02

**Authors:** Alexander E. Berezin, Tetiana A. Berezina, Evgen V. Novikov, Oleksandr O. Berezin

**Affiliations:** a Department of Internal Medicine II, Division of Cardiology, Paracelsus Medical University, 5020 Salzburg, Austria; b Department of Internal Medicine and Nephrology, VitaCenter, 69000 Zaporozhye, Ukraine; c Department of functional diagnostics, Shupyk National Healthcare University of Ukraine, Kyiv 04136, Ukraine; d Luzerner Psychiatrie AG, 4915 St. Urban, Switzerland

**Keywords:** Acutely decompensated heart failure, Acute kidney injury, Decongestion, Adropin, Biomarkers, Prognosis

## Abstract

**Background:**

Patients with acutely decompensated heart failure (ADHF) demonstrated a high risk of acute kidney injury (AKI) and its transition to acute kidney disease after diuretic therapy to reach euvolemic status. The purpose of the study was to investigate whether circulating levels of adropin predict AKI in ADHF patients after decongestive therapy.

**Material and methods:**

A total of 325 individuals fulfilling the inclusion criteria of ADHF were consecutively enrolled from October 2020 to October 2024. The study was designed as prospective cohort study. The congestion was assessed using Framingham criteria of congestion (Framingham heart failure score ≥ 2). Patients with AHDF were divided into 2 groups according to the presence of AKI (*n* = 113) and without AKI (*n* = 212). Circulating levels of N-terminal brain natriuretic pro-peptide (NT-proBNP), high-sensitivity C-reactive protein, high-sensitive troponin T, interleukin-6, tumor necrosis factor-alpha, soluble suppression of tumorigenicity-2, procalcitonin were measured. Predictors of AKI were identified using univariate and multivariate logistic regression analysis.

**Results:**

We found that the presence of atrial fibrillation, urinary albumin/creatinine ratio (UACR) ≥16.5 mg/g Cr, serum levels of adropin<2.1 ng/mL and NT-proBNP ≥19,540 pmol/mL were independent predictors for AKI in patients with ADHF. UACR and atrial fibrillation revealed a strict similarity in prediction of AKI, whereas discriminative ability of adropin<2.1 ng/mL were higher to NT-proBNP ≥19,540 pmol/mL. The combined predictive model (low levels of adropin + higher levels of NT-proBNP) showed significantly better discriminatory power compared to other models.

**Conclusion:**

Low levels of adropin<2.1 ng/mL on hospital admission in patients with ADHF can predict AKI and that its predictive ability was significantly higher compared with the conventionally used urinary albumin/creatinine ratio and NT-proBNP. Adropin may add predictive information to NT-proBNP for AKI in individuals with ADHF.

## Highlights

• Serum adropin<2.1 ng/mL were associated with acute kidney injury in patients with acutely decompensated heart failure
• Predictive ability of low levels of adropin is higher to urinary albumin/creatinine ratio
• Low serum levels of adropin improve the discriminatory power of NT-proBNP for acute kidney injury
• Measurement of adropin can prevent acute kidney injury after decongestion during hospital stay

## Graphical abstract

**Figure:**
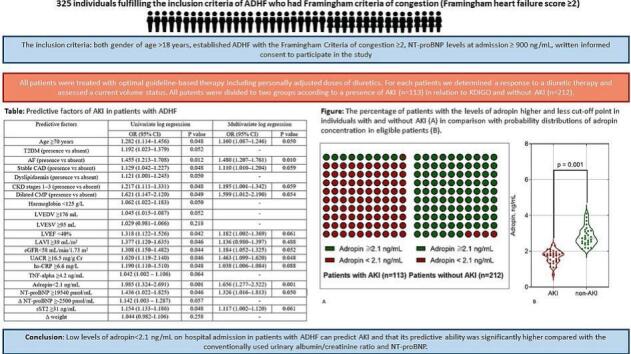

## 1 Introduction

Acute kidney injury (AKI) is common complication of acute heart failure (HF) especially after decongestion [1,2]. Although decongestion therapy is associated with improved clinical status and short-term survival among patients hospitalized with acute HF, it often interferes with a risk of renal dysfunction [3,4]. Despite strong evidence for the beneficial effects of decongestion in improving long-term renal function, AKI significantly worsens short-term prognosis and is associated with an increased risk of all-cause and cardiovascular death, prolonged hospitalization, poor quality of life, and increased economic burden [5,6].

According to the Kidney Disease: Improving Global Outcomes (KDIGO), AKI is defined as abnormalities of renal function and/or structure with an increase in serum creatinine >0.3 mg/dL (26.5 μmol/L) and lasting ≤7 days [7]. However, the pathophysiological mechanisms that contribute to the increased importance of AKI in acute HF patients on decongestive therapy are not fully understood [8]. It has been suggested that sympathoadrenal, renin-angiotensin-aldosterone (RAAS) and endothelin activation, as well as pro-inflammatory activation with increased circulating levels of inflammatory cytokines / chemokines, are associated with worsening renal perfusion due to low perfusion pressure, haemodynamic instability, decongestion and increased plasma viscosity [9,10]. It leads to endothelial dysfunction, neurohumoral and pro-inflammatory activation that can lead to vasoconstriction, platelet activation and circulating blood sludge, microvascular thrombosis and cortical/parenchymal blood flow dysregulation and critical decline in effective renal perfusion [11]. As a result, these factors exacerbate the vicious circle by further activating the local and circulating RAAS and overexpressing inflammatory cytokines, causing tubular ischaemia with tubular dysfunction, impaired regulation of the intraglomerular pressure gradient, ischaemia/necrosis of the glomeruli and, finally, a reduction in diuresis [12]. It should be noted that compression of renal structures due to limited space for expansion, known as “renal tamponade”, has recently been proposed as a novel pathophysiological factor in the association between cardiac and renal dysfunction, while its role in decongestion-induced AKI has not been widely studied [13]. In addition, several comorbidities that are highly prevalent in patients with acute HF, such as atrial fibrillation, ischemia-induced cardiomyopathy, primary or secondary mitral and tricuspid regurgitations, diabetes mellitus, rheumatic and respiratory diseases, pre-existing chronic kidney disease/end stage renal disease, may contribute to increased AKI rates and mortality through several interactions, including fluid retention, inflammation, and pulmonary embolism [14,15].

Identifying acute HF patients at higher risk of AKI and their transition to AKD after diuretic therapy is considered a promising approach to predict adverse clinical outcomes and improve in-hospital mortality [16]. Furthermore, regular diuretic use to prevent persistent congestion may correspond to the transition from AKD to CKD and increase the risk of CKD-related outcomes, whereas this association has been demonstrated in patients with moderate to severe CKD [17]. Conventionally used biomarkers of renal injury (creatinine/albumin ratio, cystatin C, neutrophil gelatinase-associated lipocalin, kidney injury molecule-1), biomechanical stress biomarkers such as N-terminal prohormone of brain natriuretic peptide (NT-proBNP) and cardiac troponins), fibrosis biomarkers (transforming growth factor [TGF]-beta-1, soluble suppression of tumorigenicity-2 [sST2]) and inflammatory biomarkers (tumor necrosis factor (TNF)-alpha, high-sensitivity C-reactive protein (hs-CRP), interleukins [IL]-1, IL-6, IL-10) have demonstrated sufficient prognostic significance in patients with acute HF with congestion [[18], [19], [20]]. Unfortunately, they could explain about 40 % of the difference in the outcome between individuals with and without decongestive-induced AKI [20]. On the other hand, there is a well-developed nomogram for predicting in-hospital mortality in heart failure patients with pre-existing CKD, but it has not been validated in acute HF patients at higher risk of AKI after decongestion [21]. In view of the above, the search for new AKI indicators with prognostic value suitable for patients with acute HF receiving decongestive therapy seems promising.

Adropin, a multifunctional protein discovered in 2008 and expressed in various tissues and cells including the kidney, heart, liver and vasculature, plays a key role in energy homeostasis and has marked anti-inflammatory, antioxidant, angiopoietic and tissue-protective properties [22]. Adropin acts as a sensitizer of insulin signalling pathways such as Akt / phosphatidylinositol-3(PI3)-kinase and Shc / the mitogen-activated protein kinase (MAPK) with further activation of the glucose transporter 4 receptor [23]. Adropin also inhibited the phosphorylation of pyruvate dehydrogenase, inhibited the insulin-signalling phosphorylation of JNK and positively influenced the activity of phosphoenolpyruvate carboxykinase-1, restoring insulin sensitivity and promoting gluconeogenesis [24]. Adropin showed a positive correlation with phosphoenolpyruvate carboxykinase-1, a key regulator of gluconeogenesis. In addition, Adropin upregulated the expression of peroxisome proliferator-activated receptor γ (PPARγ) through the enhancement of Akt/PI3 kinase, thereby promoting M2 polarisation and modulating lipid profiles [25]. However, it had no significant effect on oxidized low-density lipoprotein-induced foam cell formation in macrophages [26]. Adropin markedly suppressed the production of a broad spectrum of pro-inflammatory cytokines, including TNF-alpha and IL-6, by activating extracellular signal-regulated kinase ½ (ERK 1/2) via vascular endothelial growth factor receptor 2 (VEGFR2) [27]. Therefore, adropin was found to be an inducer of nuclear factor erythroid 2-related factor 2 (Nrf2), which is involved in down-regulating oxidative stress, attenuating tissue protection and reducing the levels of inducible reduced levels of endothelial nitric oxide synthase [28,29]. Interestingly, in an animal model, adropin increased cell viability acting through pro-survival kinases (Akt/PI3K and ERK1/2), as well as reduced apoptosis by down-regulating caspase-3 activity and Bax along with promoting Bcl-2 expression and increasing the Bcl-2/Bax ratio [30].

In clinical settings, the levels of serum adropin were inversely associated with the presence of atherosclerosis, coronary artery disease, acute myocardial infarction, heart failure, atrial fibrillation, AKI, CKD, and diabetes mellitus [[31], [32], [33], [34], [35], [36]]. Furthermore, adropin exerted its independent predictive power for CKD in individuals with chronic HF regardless of phenotype and type 2 diabetes mellitus, as well as in newly asymptomatic HF with preserved ejection fraction (HFpEF) [37,38]. Despite the significance of these findings, the precise predictive role of adropin for AKI following decongestive therapy in AHF patients remains poorly understood. The purpose of the study was to investigate whether adropin predicts AKI in acutely decompensated HF (ADHF) patients after decongestive therapy.

## 2 Material and methods

### 2.1 Patient population and study design

We selected 736 white male and female, who were admitted with a diagnosis of AHF, among whom 325 individuals fulfilling the inclusion criteria of ADHF were consecutively enrolled from October 2020 to October 2024. Then they were longitudinally evaluated during hospital stay at the private hospital “Vita Center” (Zaporozhye, Ukraine).

**Fig. 1:**
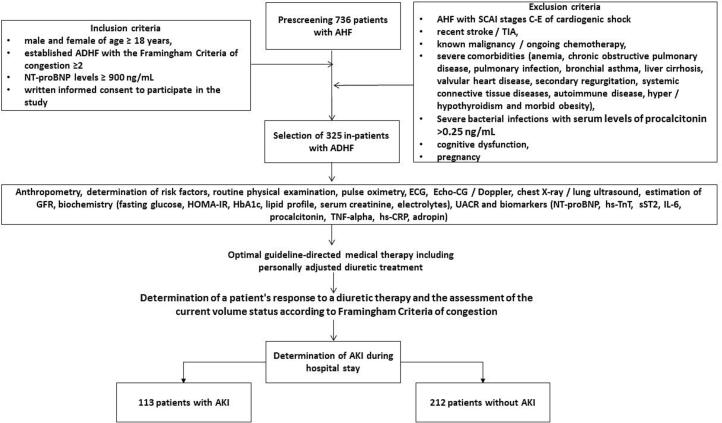
Flow chart and study design. Abbreviations: ADHF, acutely decompensated heart failure; AHF, acute heart failure; AKI, acute kidney injury; CRP, C-reactive protein; HOMA-IR, Homeostatic Assessment Model of Insulin Resistance; HbA1c, glycosylated haemoglobin; GFR, glomerular filtration rate; IL, interleukin; NT-proBNP, N-terminal brain natriuretic pro-peptide; SCAI, Society for Cardiovascular Angiography and Interventions; sST2, soluble suppression of tumorigenicity-2; TIA, transient ischemic attack; TnT, troponin T; TNF, tumor necrosis factor.

The inclusion and exclusion criteria, as well as study procedures and determination of clinical outcomes, are outlined in Fig. 1. Inclusion criteria were: both gender of age > 18 years, established ADHF with the Framingham Criteria of congestion ≥2, NT-proBNP levels at admission ≥900 ng/mL, written informed consent to participate in the study. Individuals with Society for Cardiovascular Angiography and Interventions (SCAI) stages C-E of cardiogenic shock, recent stroke / transient ischemia attack (TIA), known malignancy / ongoing chemotherapy, severe comorbidities (anaemia, chronic obstructive pulmonary disease, pulmonary infection, bronchial asthma, liver cirrhosis, valvular heart disease, secondary regurgitation, systemic connective tissue diseases, autoimmune disease, hyper / hypothyroidism and morbid obesity), severe bacterial infections with serum levels of procalcitonin >0.25 ng/mL, cognitive dysfunction and pregnancy were not included in the study.

All in-patients were treated with optimal guideline-based therapy including personally adjusted doses of diuretics. For each patients we determined a response to a diuretic therapy and assessed a current volume status. The patents were divided to two groups according to a presence of AKI (*n* = 113) and without AKI (*n* = 212).

### 2.2 Ethical approval

The study was conducted according to the guidelines of the Declaration of Helsinki and approved by the Institutional Ethics Committee of Zaporozhye Medical Academy of Post-graduate Education (protocol number: 8; date of approval: 10 October 2020). All patients gave their informed consent to the use of their personal data for scientific purposes in this study and in future studies, as they simultaneously completed the mandatory consent form for the transfer of their data to the medical institution in accordance with the current legislation on health protection and personal data retention.

### 2.3 Determination of ADHF and volume status

ADHF was defined as AHF with progressive fluid retention/systemic congestion and/or peripheral hypoperfusion [39]. Diagnosis of AHD was based on 2021 ESC criteria, which include a) rapid or gradual onset of symptoms and/or signs of HF, b) a need to urgent medical attention, c) leading to an unplanned hospital admission [39]. To define the congestion we used the Framingham Criteria [40], which exhibit high specificity for congestion and are strongly associated with HF phenotypes, elevated right atrial pressures and right ventricular (RV) dysfunction determined with tricuspid annular plane systolic excursion [41]. The patients should demonstrate two more criteria of fluid retention including jugular venous distension and hepato-jugular reflux, which are the primary criteria for defining ADHF. SCAI criteria of cardiogenic shock were used to determine stages of cardiogenic shock [42]. The assessment of the current volume status and patient's response to diuretic therapy were evaluated according to current guidelines [39,43]. Continued monitoring of patient weights during hospital admissions for volume overload secondary to HF was provided. Euvolumic status was confirmed when patients had no Framingham Criteria of congestion [44].

### 2.4 Determination of AKI

AKI was determined according to KDIGO (Kidney Disease: Improving Global Outcomes) criteria [7]. AKI is defined as the presence of any of the following: a) increase in serum creatinine by 0.3 mg/dL or more (26.5 μmol/L or more) within 48 h; b) increase in serum creatinine to 1.5 times or more the baseline value of the previous 7 days; c) urine output <0.5 mL/kg/h for at least 6 h [7].

### 2.5 Echocardiography examination

In the study, all individuals were undergone the standard transthoracic B-mode ultrasound examination that was provided by high qualified assessors in apical 2- and 4-chamber views using a GE Healthcare Vivid E95 scanner (General Electric Company, Horton, Norway). The conventional hemodynamic parameters, including cardiac dimensios, left ventricular end-diastolic and end-diastolic volumes, left atrial volum index (LAVI), tricuspid annular plane systolic excursion (TAPSE) were evaluated according to 2018 Guideline of the American Society of Echocardiography [45]. Left ventricular ejection fraction (LVEF) was determined by Simpson's method. Doppler examination was performed to determine mitral and tricuspid regurgitaions and to measure early diastolic mitral blood filling (E), medial and lateral e` velocities. The estimated E/e` ratio was expressed as the ratio of the E wave velocity to the averaged medial and lateral e` velocities. Left ventricular hypertrophy was defined as a left ventricular mass index (LVMI) ≥ 95 g/m^2^ in women or ≥ 115 g/m^2^ in men [45].

### 2.6 Glomerular filtration rate and insulin resistance determination

The conventional CKD-EPI formula was used to estimate the glomerular filtration rate (eGFR) [46]. The Homeostatic Assessment Model of Insulin Resistance (HOMA-IR) was used to assess insulin resistance [47].

### 2.7 Blood sampling and biomarker analysis

Fasting blood samples were received from the peripheral vein and collected in BD Vacutainer Serum Plus Tube. Blood samples were stored for 30 min at room temperature to clot. After clotting the samples were centrifuged at 3000 rpm for 15 min. Samples with haemolyses were not used for further evaluation. The supernatant was collected and stored at −70 °C until analysis at certified laboratory of the Vita Centre (Zaporozhye, Ukraine).

**Fig. 2:**
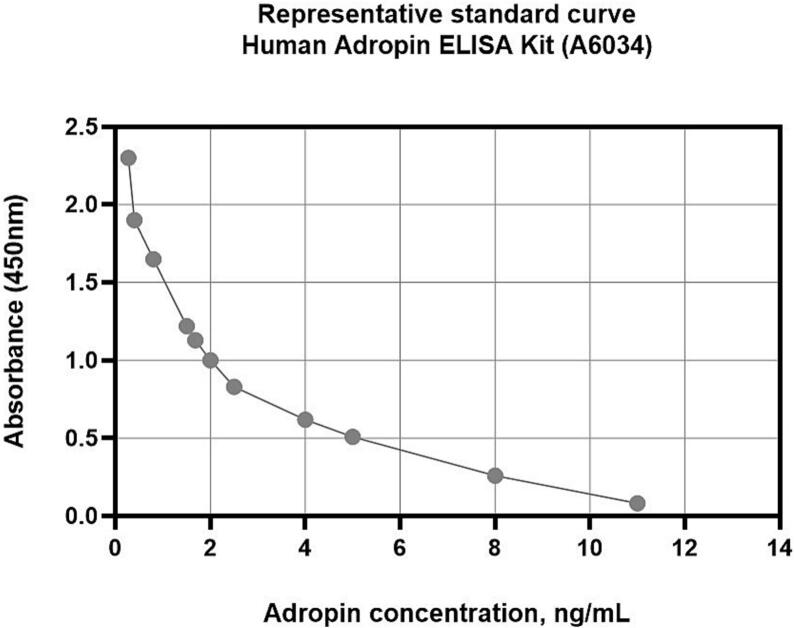
Standard curve in which the average absorbance for each standard is plotted on the Y (vertical) axis against the known standard concentrations on the X (horizontal) axis.

We measured circulating biomarkers in serum at the baseline. The levels of NT-proBNP, TNF-alpha, sST2, hs-CRP, high-sensitive troponin T, IL-6, procalcitonin were detected using ELISA kits (Elabscience, Houston, Texas, USA), adropin levels were measured with ELISA kit (Antibodies.com, Stockholm, Sweden). Analyses were performed according to the manufacturer's instructions and used as direct measurements further. Each sample was analysed twice and the average was used for the final evaluation. A standard curve were generated for each set of samples assayed. Fig. 2 illustrate the representative standard curve for Human Adropin ELISA Kit (catalog number: A6034, Stockholm, Sweden) (concentration range: 0.156–10 ng/mL). Both intra- and inter-assay coefficients of variability for each marker were < 10 %.

Conventional haematological and biochemical parameters were determined with a Roche P800 analyser (Basel, Switzerland) without freeze.

### 2.8 Statistical analysis

Statistical analysis of data was carried out using SPSS Statistics 29 (IBM, Armonk, NY, USA) and Prism v.10 (GraphPad, San Diego, CA, USA) software. Variables are reported as mean and standard deviation, median and interquartile range (IQR), or absolute numbers and percentages (%), as appropriate. The Anderson-Darling test was used to check for data distribution. Continuous variables were compared using paired *t*-test or Mann-Whitney test when appropriate, whereas categorical variables were compared using Fisher's exact test. Spearman's correlation coefficient (r) was utilized for correlations between hemodynamic parameters, comorbidities and biomarkers including the levels of adropin. The Benjamini-Hochberg procedure was utilized in order to calculate the False Discovery Rate-adjusted *P*-Value for each pair of variables. Plausible predictors of AKI were identified using univariate logistic regression and backward stepwise multivariate logistic regression. An odds ratio (OR) and 95 % confidence interval (CI) were calculated for each predictor. Predictors with a significance of *P* < 0.05 in the univariate log regression analysis were included in the multivariate log regression model. The reliability of the predictive models was determined by Receiver Operating Curve (ROC) analysis, with further calculation of area under the curve (AUC), its CI, sensitivity (Se), and specificity (Sp) for each predictor. The Youden test was used to estimate the cut-off points for adropin. We compared the incremental prognostic capacity of models on using a binary prediction methodology based on the estimation of integrated discrimination indices (IDI) and net reclassification improvement (NRI). *P*-value <0.05 was considered statistically significant.

## 3 Results

### 3.1 Basic clinical characteristics, echocardiographic parameters and laboratory findings

The mean age of the patients in this study was 68 years, and 57.8 % were male (Table 1). They had a mean body mass index of 26.4 ± 3.12 kg/m^2^, a mean waist circumference of 101.9 ± 7.30 cm. The past medical history included smoking (48.3 %), dyslipidaemia (77.8 %), hypertension (50.7 %), stable coronary artery disease (53.5 %), dilated cardiomyopathy (17.8 %), atrial fibrillation (29.5 %), abdominal obesity (28.9 %), type 2 diabetes mellitus (36.0 %), left ventricular hypertrophy (66.8 %), and chronic kidney disease grades 1-–3 grades (22.5 %). Among the patients included in the study, 31.1 %, 26.2 % and 42.8 % had HFpEF, HF with reduced ejection fraction (HFrEF) and HF with mildly reduced ejection fraction (HFmrEF), respectively. In addition, 25.8 %, 55.4 % and 18.8 % of the patents demonstrated clinical signs of II, III and IV NYHA functional classes, respectively. Patents with ADHF had mean LVEF of 48 % (37 %–58 %), enlargement of left ventricle, mean LAVI of 44 mL/m^2^ (38 mL/m^2^–51 mL/m^2^), an average TAPSE of 21 mm (15 mm −24 mm) and E/e` ratio of 15 ± 6 units. The dynamic changes of serum creatinine level with more than twofold increase are noteworthy. Along with it, the patients had moderate elevation of inflammatory cytokines, such as hs-CRP, TNF-alpha, IL-6, sST2, and marked increase in serum levels of NT-proBNP, decrease in adropin levels without sufficient changes in cTnT and procalcitonin. All individuals were optimally treated in accordance of current guideline and received renin-angiotensin-aldosterone system inhibitors in combination with beta-blockers, SGLT2 inhibitors and other drugs when needed. Patients with atrial fibrillation were treated with anticoagulants. Individuals with CAD received antiplatelet agents. Patients with type 2 diabetes mellitus were treated with metformin as initial therapy and additionally SGLT2 inhibitors or GKP-1-RAs when needed. Decongestion therapy with diuretics was provided for all occasions. At least 25 % of patients have been received vasopressors or inotropes. Resynchronized therapy was utilized in 7.4 % cases. We did not find significant differences between cohorts in terms of age, gender, anthropometric parameters, the presence of CV risk factors (smoking history, dyslipidaemia, abdominal obesity, hypertension), as well as comorbidities such as dilated cardiomyopathy, left ventricular hypertrophy, type 2 diabetes mellitus, phenotypes of HF, NYHA classes, systolic and diastolic blood pressure, LVEF, TAPSE, E/e`, HOMA-IR, lipid profile, serum levels of electrolytes, fasting glucose, haemoglobin, haematocrit, protein, creatinine, hs-CRP, TNF-alpha, NT-proBNP, sST2, cTnT, IL-6, procalcitonine and concomitant medications. However, the patients with AKI had frequently chronic coronary artery disease, atrial fibrillation, and chronic kidney disease than those without AKI. Moreover, patients with AKI demonstrated higher left ventricular end-diastolic and end-systolic volumes, LAVI, urinary albumin/creatinine ratio, creatinine levels at baseline as well as 48-h and 7-day creatinine levels, and lower eGFR and adropin than those without AKI.

**Table 1:** Baseline general characteristics of eligible patients.

Variables	Entire ADHF patient cohort (*n* = 325)	Patients with AKI (*n* = 113)	Patients without AKI (*n* = 212)	*P* value between cohorts
Age, year	68 (59–77)	69 (61–76)	68 (57–70)	0.366
Male gender, *n* (%)	188 (57.8)	67 (59.3)	121 (57.1)	0.168
BMI, kg/m^2^	26.4 ± 3.12	25.9 ± 3.04	26.7 ± 2.95	0.532
Δ weight, kg	−3 (−5–0)	−3 (−5–0)	−3 [−5–0]	–
Waist circumference, cm	101.9 ± 7.30	102.3 ± 5.10	100.8 ± 8.13	0.626

Past medical history				
Smoking history, *n* (%)	157 (48.3)	56 (49.6)	101 (47.6)	0.592
Dyslipidaemia, *n* (%)	253 (77.8)	85 (75.2)	168 (79.2)	0.188
Abdominal obesity, *n* (%)	94 (28.9)	33 (29.2)	61 (28.8)	0.722
Hypertension, *n* (%)	165 (50.7)	58 (51.3)	107 (50.5)	0.683
Stable CAD, *n* (%)	174 (53.5)	72 (63.7)	102 (48.1)	0.048
Dilated cardiomyopathy, *n* (%)	58 (17.8)	21 (18.6)	37 (17.5)	0.547
AF, *n* (%)	96 (29.5)	37 (32.7)	59 (27.8)	0.046
LVH, *n* (%)	217 (66.8)	76 (67.3)	141 (66.5)	0.643
CKD 1–3 grades, *n* (%)	73 (22.5)	31 (27.4)	42 (19.8)	0.042
T2DM, *n* (%)	117 (36.0)	43 (38.1)	74 (34.9)	0.160
HFpEF, *n* (%)	101 (31.1)	33 (29.2)	68 (32.1)	0.762
HFrEF, *n* (%)	85 (26.2)	30 (26.5)	55 (25.9)	0.644
HFmrEF, *n* (%)	139 (42.8)	50 (44.2)	89 (42.0)	0.618

NYHA functional class, *n* (%)				
II	84 (25.8)	28 (24.8)	56 (26.4)	0.407
III	180 (55.4)	61 (54.0)	119 (56.1)	0.522
IV	61 (18.8)	24 (21.2)	37 (17.5)	0.068

Hemodynamic and echocardiographic parameters				
Systolic BP, mm Hg	131 ± 10	127 ± 7	135 ± 9	0.112
Diastolic BP, mm Hg	69 ± 7	66 ± 5	73 ± 6	0.132
LVEDV, mL	169 (147–193)	178 (145–213)	161 (145–191)	0.044
LVESV, mL	87 (69–101)	94 (77–114)	81 (65–98)	0.046
LVEF, %	48 (37–58)	47 (35–57)	49 (38–59)	0.054
LVMI, g/m^2^	154 ± 19	157 ± 17	149 ± 15	0.114
LAVI, mL/m^2^	44 (38–51)	46 (40–53)	41 (35–48)	0.042
TAPSE, mm	21 (15–24)	19 (15–23)	22 (17–26)	0.547
E/e`, unit	15 ± 6	16 ± 4	14 ± 4	0.298

Laboratory findings				
Baseline eGFR, mL/min/1.73 m^2^	66 ± 15	61 ± 9	70 ± 11	0.040
UACR (mg/g Cr)	14.2 (6.5–20.4)	16.9 (8.3–22.5)	12.3 (5.1–19.6)	0.042
K, mmol/L	4.05 (3.46–4.78)	4.02 (3.32–4.65)	4.08 (3.58–4.90)	0.704
Na, mmol/L	139 (136–142)	137 (134–141)	139 (135–144)	0.612
HOMA-IR, units	5.17 ± 2.33	5.22 ± 2.10	5.07 ± 2.07	0.354
Fasting glucose, mmol/L	4.84 ± 0.69	4.89 ± 0.58	4.73 ± 0.56	0.614
HbA1c, %	5.05 ± 1.04	5.18 ± 0.96	5.06 ± 0.88	0.480
Haemoglobin, g/L	13.6 (12.7–14.5)	13.3 (12.5–14.2)	13.8 (12.6–14.9)	0.442
Haematocrit, %	37 (35–41)	38 (34–42)	36 (33–41)	0.188
Protein, g/L	68 (65–71)	67 (64–70)	68 (65–72)	0.633
Baseline creatinine, μmol/L	109 ± 11	113 ± 19	105 ± 10	0.058
48-h creatinine, μmol/L	298 ± 42	415 ± 38	119 ± 27	0.001
7-day creatinine, μmol/L	117 ± 21	129 ± 17	106 ± 15	0.001
Serum uric acid, μmol/L	365 ± 91	470 ± 98	338 ± 79	0.024
Total cholesterol, mmol/L	5.66 ± 0.82	5.69 ± 0.65	5.61 ± 0.76	0.492
HDL-C, mmol/L	1.09 ± 0.11	1.06 ± 0.10	1.11 ± 0.12	0.540
LDL-C, mmol/L	3.50 ± 0.23	3.62 ± 0.21	3.37 ± 0.20	0.426
Triglycerides, mmol/L	2.31 ± 0.25	2.37 ± 0.24	2.24 ± 0.19	0.374
hs-CRP, mg/L	6.13 (2.98–9.10)	6.72 (3.05–9.83)	5.98 (3.11–8.86)	0.620
TNF-alpha, pg/mL	3.52 (2.10–4.90)	3.70 (2.05–5.12)	3.46 (2.02–4.76)	0.477
NT-proBNP, pmol/mL	10,956 (1280–21,985)	12,450 (1170–24,587)	10,217 (1136–20,448)	0.544
Δ NT-proBNP, pmol/mL	−2917 (−4168 to −578)	−2553 (−3912 to −444)	−3257 (−4658 to −611)	0.146
sST2, ng/mL	12.25 (2.78–22.36)	12.86 (4.44–35.12)	11.74 (1.92–21.47)	0.655
cTnT, ng/mL	0.054 (0.024–0.12)	0.055 (0.018–0.13)	0.052 (0.01–0.10)	0.612
IL-6, ng/mL	2.57 (0.88–4.72)	2.61 (0.92–4.88)	2.40 (0.62–4.47)	0.540
Procalcitonin, ng/mL	0.08 (0.04–0.13)	0.09 (0.04–0.15)	0.06 (0.01–0.16)	0.618
Adropin, ng/mL	2.17 (1.70–2.64)	1.83 (1.32–2.35)	2.73 (2.20–3.15)	0.001

Concomitant medications and devises				
ACE inhibitors, *n* (%)	121 (37.2)	41 (36.3)	80 (37.7)	0.622
ARBs, *n* (%)	48 (14.8)	17 (15.0)	31 (14.6)	0.658
ARNI, *n* (%)	145 (44.6)	48 (42.5)	97 (45.8)	0.408
Beta-blockers, *n* (%)	287 (88.3)	101 (89.4)	186 (87.7)	0.657
Ivabradine, *n* (%)	28 (8.6)	9 (8.0)	19 (9.0)	0.845
CCBs, *n* (%)	39 (12.0)	12 (10.6)	27 (12.7)	0.060
MRA, *n* (%)	87 (26.8)	27 (24.9)	60 (28.3)	0.318
Diuretics, *n* (%)	325 (100.0)	113 (100.0)	212 (100.0)	–
Vasopressors / inotropes, *n* (%)	81 (24.9)	28 (24.7)	53 (25.0)	0.644
Antiplatelet agents, *n* (%)	174 (53.5)	72 (63.7)	102 (48.1)	0.048
Anticoagulants, *n* (%)	96 (29.5)	37 (32.7)	59 (27.8)	0.046
Metformin, *n* (%)	117 (36.0)	43 (38.1)	74 (34.9)	0.160
SGLT2 inhibitors, *n* (%)	219 (67.4)	75 (66.3)	144 (67.9)	0.511
GLP-1-RAs, *n* (%)	39 (12.0)	12 (10.6)	27 (12.7)	0.387
Statins, *n* (%)	253 (77.8)	85 (75.2)	168 (79.2)	0.251
RCT, *n* (%)	24 (7.4)	9 (8.0)	15 (7.1)	0.782

Notes: Variables are given as M ± SD and Me (25–75 % IQR). Chi-square test was used to compare categorical variables. Δ NT-proBNP is the difference between NT-proBNP at admission and discharge; Δ weight is the difference between weight on admission and discharge.

Abbreviations: AF, atrial fibrillation; ARBs, Angiotensin-II receptor blockers**;** ARNI, angiotensin receptor neprilysin inhibitors**;** BMI, body mass index; CAD, coronary artery disease; CCBs, calcium channel blockers; CKD, chronic kidney disease; eGFR, estimated glomerular filtration rate; E/e`, early diastolic blood filling to longitudinal strain ratio; HDL—C, high-density lipoprotein cholesterol; hs-CRP, high-sensitivity C-reactive protein; GLP-1-RAs, glucagon-like peptide-1 receptor agonists; HbAc1, glycosylated haemoglobin; HOMA-IR, Homeostatic Assessment Model of Insulin Resistance; HFpEF, heart failure with preserved ejection fraction; HFrEF, heart with reduced ejection fraction; HFmrEF, heart failure with mildly reduced ejection fraction; IL, interleukin; LDL-C, low-density lipoprotein cholesterol; LAVI, left atrial volume index; LVEDV, left ventricular end-diastolic volume; LVESV, left ventricular end-systolic volume; LVEF, left ventricular ejection fraction; LVMI, left ventricle myocardial mass index, LVH, left ventricular hypertrophy; MRA, mineralocorticoid receptor antagonist**;** RCT, resynchronized therapy; NT-proBNP, N-terminal brain natriuretic pro-peptide; TNF-alpha, tumor necrosis factor-alpha; sST2, soluble suppression of tumorigenicity-2; SGLT2, sodium-glucose co-transporter-2; UACR, urinary albumin/creatinine ratio; WHR, waist-to-hip ratio.

### 3.2 Correlation analysis for serum levels of adropin with other parameters and biomarkers of renal function

**Fig. 3:**
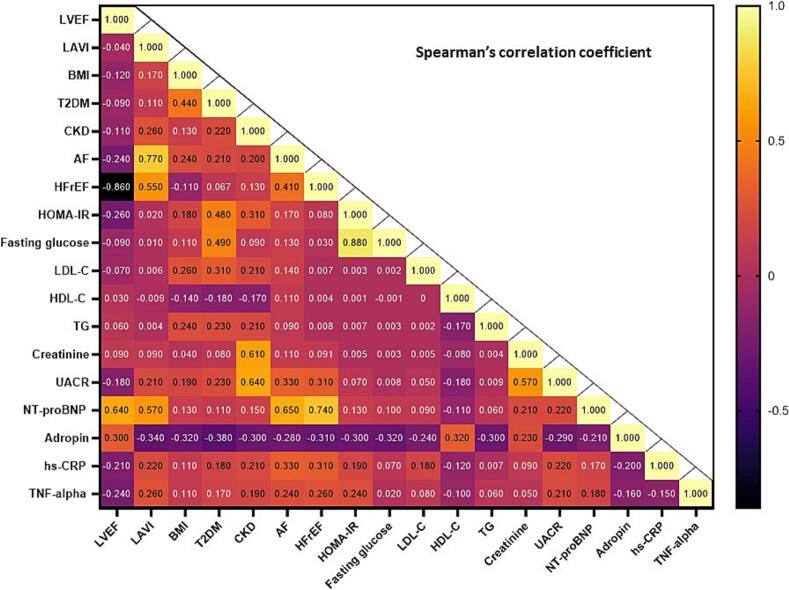
Heat map with Spearmen correlations between each pair of variables**.** Abbreviations: AF, atrial fibrillation; BMI, body mass index; CKD, chronic kidney disease; hs-CRP, high-sensitivity C-reactive protein; HOMA-IR, Homeostatic Assessment Model of Insulin Resistance; HDL-C, high-density lipoprotein cholesterol; HFrEF, heart with reduced ejection fraction; LAVI, left atrial volume index; LVEF, left ventricular ejection fraction; LDL-C, low-density lipoprotein cholesterol; NT-proBNP, N-terminal brain natriuretic pro-peptide; TG, triglycerides; TNF-alpha, tumor necrosis factor-alpha; UACR, urinary albumin/creatinine ratio.

The heat map provides a visual representation of the Spearmen correlation coefficients between each pair of variables including hemodynamic parameters, comorbidities and biomarkers (Fig. 3). Our study has revealed that the levels of serum adropin were positively correlated with LVEF (*r* = 0.30; *P* = 0.001), HDL-C levels (*r* = 0.32; *p* = 0.018), creatinine (*r* = 0.23; *p* = 0.024) and inversely with a presence of atrial fibrillation (*r* = −0.28; *P* = 0.001), CKD (*r* = −0.30; P = 0.001), T2DM (*r* = −0.38; P = 0.001), HFrEF (*r* = −0.31; P = 0.001), LAVI (*r* = −0.34; P = 0.001), BMI (*r* = −0.32; P = 0.001), fasting glucose (r = −0.32; *P* = 0.012), HOMA-IR (r = −0.30; P = 0.012), UACR (*r* = −0.29; P = 0.001), triglyceride levels (r = −0.30; *P* = 0.010), NT-proBNP(*r* = −0.21; *P* = 0.026).

### 3.3 The optimal cut-offs for possible predictors of AKI: The results of the ROC curve analysis

ROC curve analysis was performed to determine the optimal cut-offs for possible predictors of AKI (Table 2). We identified the following factors: age ≥ 70 years, haemoglobin <125 g/L, LVEDV ≥176 mL; LVESV ≥95 mL; LVEF <40 %; LAVI ≥39 mL/m^2^, eGFR <58 mL/min/1.73 m^2^; UACR ≥16.5 mg/g Cr; hs-CRP ≥6.6 mg/L; TNF-alpha ≥4.2 ng/mL; adropin <2.10 ng/mL; NT-proBNP ≥19,540 pmol/mL; sST2 ≥ 31 ng/mL, respectively.

**Table 2:** Receiver operating characteristic curve analysis for possible predictive factors of AKI in patients with ADHF.

Variables	AUC	95 % CI	P value	Cut-off	Se, %	Sp,%
Age	0.695	0.672–0.720	0.001	70 years	74.6	74.8
Haemoglobin	0.631	0.522–0.744	0.022	125 g/L	64.2	61.5
LVEDV	0.598	0.574–0.623	0.044	176 mL	61.4	62.7
LVESV	0.617	0.595–0.641	0.042	95 mL	65.8	69.2
LVEF	0.644	0.598–0.692	0.016	40 %	71.5	64.3
LAVI	0.778	0.715–0.843	0.001	39 mL/m^2^	73.4	76.8
eGFR	0.651	0.526–0.773	0.001	58 mL/min/1.73 m^2^	74.1	68.7
UACR	0.699	0.658–0.744	0.001	16.5 mg/g Cr	71.8	70.2
hs-CRP	0.751	0.720–0.785	0.001	6.6 mg/L	76.5	81.0
TNF-alpha	0.543	0.520–0.564	0.044	4.2 pg/mL	60.1	58.6
Adropin	0.848	0.799–0.948	0.001	2.10 ng/mL	84.9	72.7
NT-proBNP	0.812	0.750–0.871	0.001	19,540 pmol/mL	81.3	82.5
Δ NT-proBNP	0.658	0.610–0.699	0.001	−2500 pmol/mL	78.4	68.9
sST2	0.755	0.721–0.787	0.001	31 ng/mL	77.1	80.3
Δ weight	0.645	0.612–0.694	0.001	−2.5 kg	68.5	69.8

Notes: Δ NT-proBNP is the difference between NT-proBNP at admission and discharge; Δ weight is the difference between body weight on admission and discharge.

Abbreviations: AUC, area under curve; CI, confidence interval; eGFR, estimated glomerular filtration rate; LVEDV, left ventricular end-diastolic volume; LVESV, left ventricular end-systolic volume; LVEF, left ventricular ejection fraction; hs-CRP, high-sensitivity C-reactive protein; LAVI, left atrial volume index; Se, sensitivity; Sp, specificity; NT-proBNP, N-terminal brain natriuretic pro-peptide; sST2, soluble suppression of tumorigenicity-2; UACR, urinary albumin/creatinine ratio; TNF-alpha, tumor necrosis factor-alpha.

### 3.4 Predictive factors for AKI: Univariate and multivariate logistic regression models

Univariate logistic regression analysis showed that age ≥ 70 years, type 2 diabetes mellitus (T2DM), atrial fibrillation, coronary artery disease (CAD), dyslipidaemia, CKD stages 1–3,dilated cardiomyopathy, LVEF <40 %, LAVI ≥39 mL/m^2^, eGFR<58 mL/min/1.73 m^2^, UACR ≥16.5 mg/g Cr, hs-CRP ≥6.6 mg/L, adropin<2.10 ng/mL, NT-proBNP≥19,540 pmol/mL and sST2 ≥ 31 ng/mL were associated with AKI development (Table 3). Multivariate logistic regression analysis revealed that AF, UACR ≥16.5 mg/g Cr, adropin<2.10 ng/mL and NT-proBNP≥19,540 pmol/mL were independent predictors for AKI in patients with ADHF.

**Table 3:** Predictive factors for AKI: Univariate and multivariate log regression analysis.

Predictive factors	Univariate log regression		Multivariate log regression	
	OR (95 % CI)	P value	OR (95 % CI)	P value
Age ≥ 70 years	1.282 (1.114–1.456)	0.048	1.160 (1.087–1.246)	0.050
T2DM (presence vs absent)	1.192 (1.023–1.379)	0.052	–	
AF (presence vs absent)	1.455 (1.213–1.708)	0.012	1.480 (1.207–1.761)	0.010
Stable CAD (presence vs absent)	1.129 (1.042–1.227)	0.048	1.110 (1.010–1.204)	0.059
Dyslipidaemia (presence vs absent)	1.121 (1.001–1.245)	0.050	–	
CKD stages 1–3 (presence vs absent)	1.217 (1.111–1.331)	0.048	1.195 (1.001–1.342)	0.059
Dilated CMP (presence vs absent)	1.621 (1.147–2.120)	0.049	1.599 (1.012–2.190)	0.054
Haemoglobin <125 g/L	1.062 (1.022–1.183)	0.050	–	
LVEDV ≥176 mL	1.045 (1.015–1.087)	0.052	–	
LVESV ≥95 mL	1.029 (0.981–1.066)	0.218	–	
LVEF <40 %	1.318 (1.122–1.526)	0.042	1.182 (1.002–1.369)	0.061
LAVI ≥39 mL/m^2^	1.377 (1.120–1.635)	0.046	1.136 (0.980–1.397)	0.488
eGFR<58 mL/min/1.73 m^2^	1.308 (1.150–1.482)	0.044	1.184 (1.052–1.325)	0.052
UACR ≥16.5 mg/g Cr	1.620 (1.119–2.140)	0.046	1.463 (1.099–1.620)	0.048
hs-CRP ≥6.6 mg/L	1.190 (1.110–1.510)	0.048	1.038 (1.006–1.084)	0.088
TNF-alpha ≥4.2 ng/mL	1.042 (1.002–1.106)	0.064	–	
Adropin<2.1 ng/mL	1.985 (1.324–2.691)	0.001	1.656 (1.277–2.522)	0.001
NT-proBNP ≥19,540 pmol/mL	1.436 (1.022–1.825)	0.046	1.326 (1.016–1.813)	0.050
Δ NT-proBNP ≥ − 2500 pmol/mL	1.142 (1.003–1.287)	0.057	–	
sST2 ≥ 31 ng/mL	1.154 (1.133–1.186)	0.048	1.117 (1.002–1.120)	0.061
Δ weight	1.044 (0.982–1.106)	0.258	–	

Notes: Δ NT-proBNP is the difference between NT-proBNP at admission and discharge; Δ weight is the difference between body weight on admission and discharge.

Abbreviations: AF, atrial fibrillation; CAD, coronary artery disease; CI, confidence interval; eGFR, estimated glomerular filtration rate; LVEDV, left ventricular end-diastolic volume; LVESV, left ventricular end-systolic volume; LVEF, left ventricular ejection fraction; hs-CRP, high-sensitivity C-reactive protein; CKD, chronic kidney disease; LAVI, left atrial volume index; NT-proBNP, N-terminal brain natriuretic pro-peptide; sST2, soluble suppression of tumorigenicity-2; OR, odds ratio; UACR, urinary albumin/creatinine ratio; TNF-alpha, tumor necrosis factor-alpha; T2DM, type 2 diabetes mellitus.

### 3.5 Comparison of the predictive models

We compared the predictive models for clinical outcome and established that the model 1 (UACR ≥16.5 mg/g Cr) and the model 2 (AF) did not differ each another in prediction of AKI, whereas the model 3 (NT-proBNP≥19,540 pmol/mL) and the model 4 (adropin<2.10 ng/mL) were significantly better than the reference value of the model 1 (Table 4). Along with it, the model 4 (adropin<2.1 ng/mL) alone was superior to Model 3 (NT-proBNP≥19,540 pmol/mL). The combined model (Model 3 + Model 4) showed significantly better discriminatory power compared to the Model 1 and other models (*P* < 0.02 for all cases).

**Table 4:** The comparisons of predictive models for AKI.

Predictive Models	Dependent Variable: AKI					
	AUC		NRI		IDI	
	M (95 % CI)	*p* value	M (95 % CI)	*p* value	M (95 % CI)	*p* value
Model 1 (UACR ≥16.5 mg/g Cr)	0.699 (0.658–0.744)	–	Reference	–	Reference	–
Model 2 (AF)	0.702 (0.685–0.728)	0.462	0.15 (0.12–0.18)	0.118	0.19 (0.14–0.26)	0.088
Model 3 (NT-proBNP≥19,540 pmol/mL)	0.812 (0.750–0.871)	0.012	0.24 (0.18–0.31)	0.046	0.36 (0.29–0.42)	0.042
Model 4 (adropin<2.1 ng/mL)	0.848 (0.799–0.948)	0.001	0.33 (0.30–0.39)	0.026	0.46 (0.40–0.51)	0.012
Model 3+ Model 4	0.911 (0.879–0.955)	0.022	0.51 (0.42–0.60)	0.001	0.55 (0.44–0.67)	0.001

Abbreviations: AUC, area under curve; CI, confidence interval; IDI, integrated discrimination in-dices; NRI, net reclassification improvement; UACR, urinary albumin/creatinine ratio; NT-proBNP, N-terminal brain natriuretic pro-peptide. Note: p value indicates a significant difference in terms of Model 1.

### 3.6 Reproducibility of adropin in comparison with NT-proBNP

**Fig. 4:**
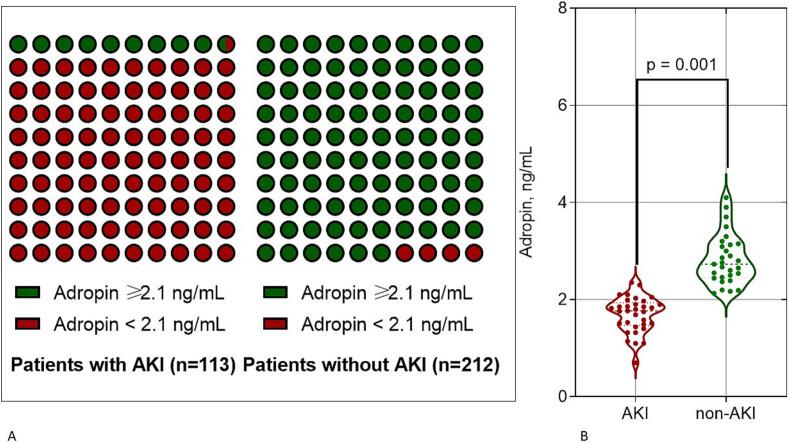
The percentage of patients with the levels of adropin higher and less cut-off point in individuals with and without AKI (A) in comparison with probability distributions of adropin concentration in eligible patients (B).

We evaluated the reproducibility of adropin compared with NT-proBNP in patients with and without AKI. The intra-class correlation coefficient for inter-observer reproducibility of NT-proBNP was 0.78 (95 % CI = 0.72–0.85). The intra-class correlation coefficient for intra-observer reproducibility of adropin was 0.87 (95 % CI = 0.81–0.93). We did not find significant changes in intra-observer reproducibility of adropin according to sex in eligible patients (*p* = 0.446). Fig. 4 shows that the only 9.7 % patients with AKI had the levels of ≥2.10 ng/mL, whereas among individuals without AKI 96.2 % of them exhibited the levels of adropin higher than the predictive cut-off.

## 4 Discussion

The study showed that the risk of AKI among patients with ADHF who were undergone with decongestion during hospital stay may be independently predicted by a reduced serum level of adropin. Furthermore, its predictive ability was found to be higher when compared with a standard approach based on UACR and NT-proBNP measurement. In addition, low levels of adropin added predictive information to elevated NT-proBNP and sufficiently improved a discriminative potency of the entire model for AKI in ADHF patients. As there are no guidelines for managing the decline in renal function that occurs with the initiation and titration of diuretics during the management of ADHF, these findings may be useful in tailoring an adaptive strategy to prevent AKI and optimise the timing of decongestion.

To the best of our knowledge, previous clinical studies have shown the potential of indices of insulin resistance, inflammatory cytokines, biomarkers of kidney injury and natriuretic peptides to predict AKI in critically ill patients with acute / acutely decompensated HF [[48], [49], [50], [51]]. While monitoring of creatinine levels is generally recommended to detect AKI and its progression to AKD, the majority of these biomarkers showed robust associations with the incidence of AKI and poor renal outcomes in critically ill HF patients [52]. However, predictive potencies of serum creatinine, natriuretic peptides, electrolytes, haematocrit retained to be uncertain in ADHF patients with lower risk and positive respond to decongestion [52,53].This is particularly important for patients hospitalized with ADHF and receiving optimal guideline-based HF therapy, including SGLT2 inhibitors, ARNI, beta-blockers and MRA, while these medications are frequently withheld during AKI because of concerns about worsening GFR and persistence of elevated creatinine [54,55].

We included in the study in-patients with ADHF who had positive response to decongestion, which was associated with a reduction of body weight during hospitalization (mean Δ weight = −3 kg**),** mean haematocrit of 37 % and consequently ΔNT-proBNP of −2917 pmol/mL (−4168 to −578). Although we included a significant number of possible predictors in the univariate logistic regression model, including age, weight and its changes, haemodynamic parameters and comorbidities, parameters of haemoconcentration, biomarkers of renal injury, biomechanical stress, myocardial injury and fibrosis, inflammatory response, concomitant medications, only a few of them were found to be associated with AKI. Indeed, old age (≥70 years), the presence of T2DM, atrial fibrillation, CAD, dyslipidaemia, CKD stages 1–3, dilated cardiomyopathy, as well as some echocardiographic parameters (LVEF <40 %, LAVI ≥39 mL/m^2^) and biomarkers (eGFR<58 mL/min/1.73 m^2^, UACR ≥16.5 mg/g Cr, hs-CRP ≥6.6 mg/L, adropin<2.1 ng/mL, NT-proBNP≥19,540 pmol/mL and sST2 ≥ 31 ng/mL) were associated with AKI development. It could be hypothesised that mentioned above comorbid conditions having an adverse effect on GFR through haemodynamic and non-haemodynamic (including inflammatory) factors would be independently associated with AKI in patients with ADHF. On the other hand, haemodynamic parameters indicative of reduced cardiac contractility and volume overload could also be associated with the risk of AKI since such patients usually require higher doses of diuretics and prolonged decongestion.

There is limited research to investigate the predictive value of various biomarkers for AKI among patients with ADHF. Some studies with small sample sizes have identified venous congestion and severe hypoperfusion as the most important risk factors for the development of AKI in ADHF patients with symptoms of cardiogenic shock [56,57]. In our study, we found that hypervolemia was strongly associated with the risk of in-hospital AKI, even in the absence of advanced stages of cardiogenic shock. However, in contrast to these studies, we were able to demonstrate that decongestion is specifically the AKI risk factor that links intensive ADHF therapy to renal injury. This requires further investigation of the predictive value of biomarkers routinely used to assess risk and severity of AKI (predominantly UACR and serum creatinine) in clinical settings other than decongestive therapy [58,59] and discovery of new biomarkers to improve discriminative potency of the models for AKI.

In fact, in multivariate regression model atrial fibrillation, UACR ≥16.5 mg/g Cr, adropin<2.1 ng/mL and NT-proBNP≥19,540 pmol/mL independently predicted AKI in patients with ADHF. Surprisingly, there was no evidence regarding possible predictive role of dynamic changes of weight and NT-proBNP. Previous studies have shown that elevated NT-proBNP independently predicted AKI in HF with any phenotypes and that reduced LVEF was another predictive factor for hemodynamically stable patients with HFrEF and HFmrEF, but not for HFpEF [60,61]. In another study, elevated circulating levels of NT-proBNP >450 pg/mL were an independent predictor of AKI in elderly patients undergoing non-cardiac surgery [62]. Yet, sST2 may be a promising biomarker for AKI in diuretic resistance [63]. However, in our study we did not find strong evidence for a plausible predictive role of inflammatory and fibrotic biomarkers, such as sST2, IL-6, TNF-alpha, hs-CRP, for AKI, whereas significantly elevated levels of NT-proBNP were associated with AKI, but decreasing levels were not. In contrast, we found that low levels of adropin alone and added to NT-proBNP were independent predictors of AKI in ADHF patients. Thus, low levels of adropin were better than NT-proBNP at predicting AKI in ADHF. Recently, we identified low levels of adropin (< 2.43 ng/mL) as an independent predictor of contrast-induced AKI in individuals with non-morbid obesity [34], but the strong discriminative power of adropin for AKI in ADHF patients requiring decongestion was first demonstrated in our study.

Indeed, low circulating levels of adropin, which has potent powerful organ-protective properties, are associated with the presence of cardiovascular and metabolic risk factors, such as atherosclerosis, hypertension, obesity, insulin resistance, chronic kidney disease and dyslipidaemia. It is likely that ADHF-related hypoperfusion of remote organs, such as liver, skeletal muscle and kidney, as well as volume overload, microvascular dysfunction, potentiating hypoxic and ischaemic changes act as triggers for decreased synthesis and release of adropin. This results in the formation of adropin deficiency, in which the peptide is unable to fully realise its effect on the main intracellular signalling activities (c-Jun N-terminal kinase, cAMP activated protein kinase A, cAMP-responsive element-binding protein) in myocardium, liver, skeletal muscles and kidney, which are involved in insulin-mediated regulation of glucose homeostasis, suppression of inflammation and oxidative stress, endoplasmic reticulum and mitochondrial stress [64]. Finally, reduced adropin levels appear to be an indicator of global metabolic, immune and inflammatory dysregulation, which strongly correlates with the severity of adverse cardiac remodelling, NYHA HF class and HF-related outcomes [[65], [66], [67]]. In this context, the evidence for a possible involvement of adropin deficiency in the development of AKI during decongestion, via a possible increased susceptibility to metabolic dysregulation, seems promising for optimising diuretic therapy regimens in ADHF.

Various methodological approaches have now been proposed to minimise the adverse effects of hypovolaemia and haemoconcentration on the risk of AKI, including monitoring NT-proBNP levels, markers of renal injury, creatinine elevation, and exclusively clinical features of congestion and response to diuretics [39,43,44]. Nevertheless, our study showed that traditional approaches were not fully effective in predicting AKI, whereas peak adropin concentration measured in addition to NT-proBNP significantly improved the overall predictive value of the model. Overall, the subject mechanisms by which adropin is able to enhance the predictive value of natriuretic peptides, markers of renal injury for AKI during decongestion remain to be explored in detail. However, already preliminary results from this study suggest a comorbid condition-independent prognostic potential of adropin for AKI during intensive diuretic therapy.

### 4.1 Study limitations

However, the study has several limitations. First, the decongestive treatment on admission was optimized in relation to the patients' clinical status, vital parameters, blood pressure and hemodynamics, and was therefore adjusted for eligibility factors and response to therapy. We did not include patients with acute HF due to myocardial infarction, acute myocarditis, systemic thromboembolism, while ischemia-induced and non-ischemia-induced cardiac dysfunction was identified as the primary cause of ADHF, along with the presence of metabolic diseases such as T2DM. However, dynamic changes in NT-proBNP and continuous monitoring of haemoconcentration together with assessment of echocardiographic parameters ensured an adaptive management regime with a lower risk of severe AKI and its progression to AKD and CKD. Secondarily, tolvaptan was not used in treatment as this agent was not approved for treatment in the country where the study was conducted. Another limitation of this study is that we did not investigate the risks of recurrent AKI in patients after discharge over an extended period. This will undoubtedly be the subject of future studies. Although many biomarkers of AKI exist and are worthy of investigation for risk assessment of AKI following decongestion, we chose to focus on common biomarkers to bring the study data closer to the realities of the clinical practice. Additionally, the study was based on including the relatively small cohort of the patients admitted to a single center. All these may require a validation of the study results. Nevertheless, the lack of mechanistic investigation can be considered as another study limitation. We believe that these limitations will not have a significant impact on the perception of our study results and their interpretation.

## 5 Conclusion

We found that serum levels of adropin<2.1 ng/mL on hospital admission in patients with ADHF can predict AKI during hospital stay and that its predictive ability was significantly higher compared with the conventionally used urinary albumin/creatinine ratio and NT-proBNP. Furthermore, decreased serum levels of adropin improved the discriminatory power of NT-proBNP for AKI in ADHF. These findings can be used to tailor decongestive therapy in people with ADHF to prevent AKI and to optimise its management.

## CRediT authorship contribution statement

**Alexander E. Berezin:** Writing – review & editing, Writing – original draft, Validation, Supervision, Project administration, Investigation, Formal analysis, Conceptualization. **Tetiana A. Berezina:** Writing – review & editing, Writing – original draft, Investigation, Formal analysis, Data curation. **Evgen V. Novikov:** Writing – review & editing, Writing – original draft, Visualization, Software, Investigation. **Oleksandr O. Berezin:** Writing – review & editing, Writing – original draft, Visualization, Software, Project administration, Investigation, Formal analysis, Data curation.

## Informed consent statement

Written informed consent has been obtained from all patients in this study.

## Institutional review board statement

This study was conducted according to the guidelines of the Declaration of Helsinki and was approved by the Institutional Ethics Committee of Zaporozhye Medical Academy of Post-graduate Education (protocol number: 8; date of approval: 10 October 2020).

## Prior publication/prior presentation

The article was not elsewhere published.

## Declaration of Generative AI and AI-assisted technologies in the writing process

The authors did not use generative artificial intelligence and artificial intelligence-assisted technologies in the writing process.

## Funding source

This research received no external funding.

## Declaration of competing interest

The authors declare that they have no known competing financial interests or personal relationships that could have appeared to influence the work reported in this paper.
